# Quitting behavior during the tobacco sales ban in South Africa: Results from a broadly nationally representative survey

**DOI:** 10.18332/tid/168594

**Published:** 2023-08-07

**Authors:** Corné van Walbeek, Robert Hill, Samantha Filby

**Affiliations:** 1 Research Unit on the Economics of Excisable Products, School of Economics, University of Cape Town, Rondebosch, South Africa; 2 Development Policy Research Unit, School of Economics, University of Cape Town, Rondebosch, South Africa

**Keywords:** COVID-19, smoking prevalence, tobacco sales ban, South Africa

## Abstract

**INTRODUCTION:**

In response to COVID-19, the South African government banned the sale of tobacco products for 20 weeks. Before the ban, the illicit cigarette market was well-entrenched and smoking cessation services were not widely available. Several surveys conducted to ascertain cigarette smokers’ responses to the ban reported substantial differences in the proportion of smokers who quit. This study provides a broadly nationally representative ex-post investigation into cigarette smokers’ quitting behavior related to the sales ban.

**METHODS:**

We used data from wave three of NIDS-CRAM (the National Income Dynamics Study-Coronavirus Rapid Mobile Survey) conducted in November–December 2020. We first investigated the proportion of people who quit and who continued smoking during and after the sales ban. We subsequently linked the NIDS-CRAM survey to the fifth wave of NIDS (2017) to identify a subset of established smokers, and considered whether their quitting behavior differed from that of all smokers who smoked at the start of the sales ban.

**RESULTS:**

The cross-sectional analysis showed that 7.8% of cigarette smokers quit during the sales ban, but that 55% of these quitters relapsed after it was lifted. Of the pre-ban smokers, 3.5% indicated that they did not smoke both during and after the sales ban, and 3.7% quit after the ban was lifted. The longitudinal analysis showed that 7% of people who were smoking in 2017, quit smoking cigarettes during the tobacco sales ban, but that >70% of quitters relapsed after it was lifted. Only 2% of pre-ban established smokers indicated that they did not smoke during or after the ban.

**CONCLUSIONS:**

The sales ban did not have the intended objective of encouraging large-scale smoking cessation. This reflects policy failures to provide smokers with appropriate cessation support and to effectively control the illicit market both prior to and during the sales ban.

## INTRODUCTION

On 15 March 2020, the South African government announced a national state of emergency in response to the COVID-19 pandemic. On 23 March 2020, the President announced a national lockdown, to start on 27 March 2020. During the lockdown the sale of all ‘non-essential’ products was banned^1^. On 25 March 2020, two days before the country went into lockdown, tobacco products were declared ‘non-essential’. Initially the lockdown restrictions were meant to last for three weeks, but after two weeks they were extended by an additional two weeks. Subsequently, restrictions on other activities were gradually lifted, but the tobacco sales ban remained in place^1^. The tobacco sales ban officially ended on 18 August 2020.

The government’s rationale for the tobacco sales ban was firmly based on promoting health and protecting the healthcare system^2^. Even though the relationship between smoking and COVID-19 was not yet established, the ban was based on the premise that smokers, given their compromised lungs, would be more susceptible to contracting COVID-19 and being hospitalised^3^. This would place unnecessary pressure on the healthcare system. The tobacco sales ban was also rationalized on the grounds that it would eliminate trips to shops to purchase tobacco products, thus reducing opportunities for COVID-19 transmission. Because the act of smoking involves the smoker regularly touching his or her face, the sales ban was also expected to reduce this possible avenue of transmitting the virus^4,5^. Subsequently, the ban was rationalized on the grounds that smokers tend to smoke in groups and share individual cigarettes, which would spread the virus through saliva^3^. The government did not provide smokers with any information about pharmacological, counselling, or any other forms of cessation support during the tobacco sales ban.

Even before the sales ban, the illicit trade in cigarettes was a serious problem in South Africa^6^. Whereas the multinational tobacco companies (British American Tobacco, Philip Morris, and Japan Tobacco International) sold most of their cigarettes through formal outlets, most of the local manufacturing companies sold their cigarettes through informal retail outlets. Many cigarettes sold in the informal sector were sold so cheaply that it is impossible that all the taxes could have been paid on them. Independent estimates (i.e. not conducted by the tobacco industry) indicate that the illicit market comprised >30% of the total market in 2017^6,7^. Tax revenue statistics indicate that, between 2017 and 2019, the illicit market decreased marginally^8^. A meeting of various government departments and the South African revenue collecting authority, held in November 2019, acknowledged that some progress had been made, but that illicit trade remained a major concern^9^.

The tobacco sales ban was comprehensive. No retail outlets, including those trading online, were allowed to sell any tobacco products or electronic nicotine and non-nicotine delivery systems. Throughout the sales ban period, the South African media regularly reported that cigarettes were freely available on the illicit market, albeit at highly elevated prices. From the beginning of May 2020 manufacturers were allowed to produce cigarettes for export, even though the sale of tobacco products in South Africa was still prohibited.

A number of surveys were conducted during the sales ban period to ascertain how people responded to the ban^10-15^. A recent evaluation of the subset of these studies that explicitly focused on quitting behavior during the sales ban^10-14^ indicated that there were substantial differences in the findings with regard to public support for the sales ban and the proportion of cigarette smokers who quit as a result of the ban^16^. Two studies claimed to be nationally representative, but considered the sales ban as part of a broader study of the impact of the lockdown on outcomes in South Africa^10,11^, whereas the two studies that looked at the tobacco sales ban in detail, were not nationally representative^12,13^.

Between April 2020 and May 2021, a team of social science researchers from various South African universities conducted five broadly nationally representative telephone-based longitudinal surveys, aimed at quantifying the impact of COVID-19 and the associated lockdown regulations on a variety of social and economic indicators. This survey, known as NIDS-CRAM (National Income Dynamics Study – Coronavirus Rapid Mobile Survey) asked respondents about (un)employment, poverty, school attendance, the prevalence of hunger and domestic violence, and attitudes towards the disease. The Research Unit on the Economics of Excisable Products (REEP), which had already conducted two online surveys of smokers during the sales ban^12,13^, approached the NIDS-CRAM organizers and were subsequently invited to formulate a number of smoking-related questions for the third round of the NIDS-CRAM survey. The survey was conducted in November and December 2020.

In this study, we report on the results of this survey, particularly as they pertain to cigarette quitting behavior during the sales ban in South Africa. The ban was controversial in South Africa, even within the tobacco-control community. Some supported the ban^16,17^, while others argued that the costs associated with the ban outweighed the benefits^12-14,18^. By presenting broadly nationally representative data for smoking and quitting behavior during the sales ban, this study hopes to address the criticism that some previous surveys were not sufficiently representative^16^ and to provide more clarity on the impact of the sales ban on quitting behavior.

## METHODS

The primary data source is wave 3 of NIDS-CRAM. NIDS-CRAM aims to measure the income, employment and welfare effects of the restrictions imposed by government to reduce the spread of the COVID-19 pandemic^19^. The sampling frame for NIDS-CRAM is based on wave 5 (2017) of the National Income Dynamics Study (NIDS), South Africa’s first nationally representative household panel study^19^. The NIDS-CRAM survey is designed to be nationally representative, just as NIDS was designed to be nationally representative. The first NIDS survey was conducted in 2008, and was followed up by waves in 2010–2011, 2012, 2014–2015, and 2017. The adult questionnaire was answered by all respondents aged ≥15 years in 2017. By the time the third wave of the NIDS-CRAM survey was conducted in November–December 2020, the youngest respondent was aged 18 years.

NIDS-CRAM is broadly nationally representative of the South African adult population (aged ≥18 years). It is broadly, rather than fully representative, for reasons such as non-random attrition in the various waves of NIDS, and unmeasurable changes in the population since the last census in 2011. Despite this, the credibility of NIDS and NIDS-CRAM has been well established, and NIDS-CRAM is generally regarded as the definitive South African survey on all COVID-related matters^20,21^.

The two most comprehensive studies that analyzed the cigarette sales ban have relied on individuals self-selecting into the sample of respondents, which could easily introduce selection bias to the results. The sampling design of the NIDS-CRAM overcomes this limitation^a^.

For NIDS-CRAM Wave 3, 6130 adults (aged ≥18 years) were interviewed. The interviews were conducted by phone. The questionnaire was designed to take a maximum of 20 min to complete. After being made aware of NIDS-CRAM, REEP approached the NIDS-CRAM team and asked them to include several tobacco-related questions in Wave 3. The request was granted, subject to the requirement that these questions should not take more than 60 s to complete. This meant that we were very limited in the number and complexity of questions that we could include.

The data for the third wave of NIDS-CRAM were collected between 2 November and 13 December 2020. For the smoking module, respondents were first asked whether they smoked cigarettes before the sales ban. If they answered no, no further questions were asked. If they answered yes, they were asked several follow-up questions. The first question was: ‘How many cigarettes did you typically smoke per day during the sales ban?’, and respondents were required to give a number. Respondents who indicated zero are regarded as having quit during the sales ban. Respondents who indicated that they ‘don't know’, were classified as continuing smokers, based on the reasoning that, even if they do not know exactly how many cigarettes they smoked, they certainly knew that they smoked. People who did not smoke cigarettes during the sales ban would certainly know that and would therefore answer ‘zero’. The tobacco module concluded with the question: ‘How many cigarettes did you typically smoke in the past week?’. By the time the survey was conducted, the sales ban had been lifted for more than ten weeks, giving respondents enough time to readjust to an environment of legal cigarette sales. Respondents who indicated that they had smoked zero cigarettes in the week before the survey are regarded as quitters. Respondents who indicated that they smoked zero cigarettes during the sales ban, but a positive number of cigarettes after the ban, are regarded as having relapsed. The questionnaire for wave 3 of NIDS-CRAM is available at https://cramsurvey.org/wp-content/uploads/2021/07/NIDS-CRAM-Wave3-Questionnaire.pdf

We focused the questions on cigarette smoking, because in South Africa the vast majority of tobacco products are consumed as cigarettes. Space and time constraints prevented us from asking questions about other tobacco products. We asked respondents to indicate the cigarette brand that they smoked during and after the sales ban. From the ‘other… please specify’ category, it was clear that some respondents smoked non-cigarette tobacco/nicotine products, including self-planted tobacco, roll-your-own tobacco, snuff, chewing tobacco, and e-cigarettes. In presenting the results, we indicate the users of these non-cigarette products in a separate category.

In the first part of the results section, we analyze smoking behavior during the sales ban period, using weighted data, based on the 2020 NIDS-CRAM survey. Specifically, we focus on the percentage of pre-ban smokers who quit during the sales ban and relapsed after the ban was lifted. In the second part of the results section, we exploit the longitudinal aspects of the NIDS dataset and link the 2020 NIDS-CRAM survey, through the unique personal identifier, to the 2017 NIDS wave 5 survey. This allows us to investigate the behavior of established smokers. We define ‘established smokers’ as individuals who indicated that they smoked cigarettes in 2017 and in 2020, before the introduction of the national sales ban.

### Statistical analysis

This study presents descriptive statistics, weighted to be broadly nationally representative of the South African population. All estimates are weighted using NIDS-CRAM sampling weights and are corrected for complex survey design as advised in the data user guide^19^. For Tables 1 and 2, we calculate relevant prevalence statistics, together with 95% confidence intervals, before, during and after the sales ban. In Table 2, two-tailed Wald tests for equivalence of means across periods were also conducted, with p-values less than 0.1, 0.05, and 0.01, indicated with one, two and three stars, respectively. For these two tables, the relevant base population is the total population aged ≥18 years, as at 2020 (i.e. the full NIDS-CRAM sample). For Table 3, the weighted number of smokers who were smoking in 2017 and 2020 formed the base for the relevant prevalence statistics. The 95% confidence intervals for all tables were calculated using standard errors corrected for the NIDS-CRAM complex survey design, which included clustering at the level of pre-defined clusters provided by Statistics South Africa, and stratification at the district council level. All statistical analyses were conducted using STATA statistical software, version 17.

**Table 1:** Estimates of smoking prevalence (%) during and after the sales ban of 2020, South Africa

	*Sample size n^a^*	*Percentage of the total adult population^b^ % (95% CI)*	*Percentage of the number of smokers before lockdown*
**Smokers before the sales ban**	737	16.9 (14.3–19.4)	100
**During the sales ban**			
Continuing cigarette smokers	647	15.3 (12.8–17.8)	90.5
Did not smoke during the sales ban (i.e. quitters)	76	1.3 (0.8–1.8)	7.8
Smoked other tobacco products during the sales ban^c^	8	0.1 (0.0–0.3)	0.9
Refused to answer	6	0.1 (0.0–0.3)	0.9
**After the sales ban**			
Continuing cigarette smokers	650	15.4 (12.9–17.8)	90.8
Did not smoke after the sales ban (i.e. quitters)	70	1.2 (0.8–1.7)	7.2
Smoked other tobacco products after the sales ban^c^	15	0.3 (0.1–0.6)	1.9
Refused to answer	2	0.0 (0.0–0.0)	0.1
**Other information**			
Quit during the ban, but smoked after the sales ban (i.e. relapsers)	41	0.7 (0.4–1.1)	4.3
Smoked during the sales ban, but quit after the sales ban ended	34	0.6 (0.3–1.0)	3.7
Quit during the sales ban and remained abstinent after the sales ban ended	35	0.6 (0.3–0.9)	3.5

a Total number of valid responses in the NIDS-CRAM is 6116 (smokers and non-smokers), representing the full adult population.

b All estimates are weighted using NIDS-CRAM sampling weights and are corrected for complex survey design.

c Although the survey was aimed only at cigarette smokers, some respondents indicated in the questions on cigarette brands purchased, that they smoked non-cigarette tobacco products during or after the sales ban. These products included self-planted tobacco, roll-your-own tobacco, chewing tobacco, snuff, and e-cigarettes.

**Table 2:** Smoking prevalence (%) by sex before, during and after the tobacco sales ban of 2020, South Africa

	*Sample n*	*Weighted population*	*Before sales ban % (95% CI)*	*During sales ban^a^ % (95% CI)*	*After sales ban % (95% CI)*
Males	2381	17257161	28.0 (24.4–31.6)	25.6 (22.0–29.2)***	25.7 (22.0–29.3)
Females	3735	19312396	6.9 (4.7–9.1)	6.1 (3.9–8.3)***	6.2 (4.3–8.0)
Total	6116	36569557	16.9 (14.3–19.4)	15.3 (12.8–17.8)***	15.4 (12.9–17.8)

a The significance stars in this column test whether the estimate of smoking prevalence during the sales ban differs significantly from the estimate of smoking prevalence before the sales ban;

*** p<0.01.

**Table 3:** Estimates of smoking prevalence among established smokers during and after the tobacco sales ban of 2020, South Africa

	*Sample size n*	*Percentage of the number of smokers before lockdown^a^ % (95% CI)*
**Number of 2017 smokers who were still smoking in 2020 (i.e. established smokers)**	480	100
**During the sales ban**		
Continuing cigarette smokers	432	91.5 (87.7–95.2)
Did not smoke during the sales ban (i.e. quitters)	40	7.0 (3.6–10.3)
Smoked other tobacco products during the sales ban^b^	6	1.0 (0.0–2.1)
Refused to answer	2	0.6 (0.0–1.6)
**After the sales ban**		
Continuing cigarette smokers	441	94.5 (91.9–97.1)
Did not smoke after the sales ban (i.e. quitters)	31	4.5 (2.1–6.9)
Smoked other tobacco products after the sales ban^b^	8	1.0 (0.1–2.0)
Continuing cigarette smokers	441	94.5 (91.9–97.1)
**Other information**		
Quit during the ban, but smoked after the sales ban (i.e. relapsers)	26	5.0 (2.0–8.0)
Smoked during the sales ban, but quit after the sales ban ended	16	2.4 (0.7–4.0)
Quit during the sales ban and remained abstinent after the sales ban ended	14	2.0 (0.3–3.7)

a All estimates are weighted using NIDS-CRAM sampling weights and are corrected for complex survey design.

b Although the survey was aimed only at cigarette smokers, some respondents indicated in the questions on cigarette brands purchased, that they smoked non-cigarette tobacco products during or after the sales ban. These products included self-planted tobacco, roll-your-own tobacco, chewing tobacco, snuff, and e-cigarettes.

## RESULTS

For NIDS-CRAM Wave 3, 6130 respondents were successfully interviewed, of which 6116 indicated their smoking status before the start of the sales ban. Of these, 737 indicated that they had smoked cigarettes in the week before lockdown. Accounting for the complex survey design, and using the weights included in the NIDS-CRAM public release, this translates to a smoking prevalence of 16.9% (95% CI: 14.3–19.4). This figure is slightly lower than previously estimated prevalence values (19.9% in 2017) for South Africa^6^. However, since we only use this as a baseline for comparison purposes, we do not make any adjustments to this estimate.

The number of sample observations, together with the weighted proportions, for the most important descriptive statistics are shown in Table 1. The discussion below refers to the weighted numbers; 95% confidence intervals are shown in parentheses in the table but are not repeated in the discussion.

Of the population of pre-lockdown cigarette smokers, 90.5% indicated that they continued smoking cigarettes during the ban, 7.8% indicated that they quit, 0.9% indicated that they smoked non-cigarette products, and 0.9% refused to answer the question.

Between the lifting of the sales ban in August 2020 and the NIDS-CRAM interview in November and December 2020, approximately 55% of smokers (4.3%/7.8%) who had quit smoking during the sales ban, had relapsed. Only 3.5% of pre-ban smokers indicated that they were not smoking both during the sales ban and after the sales ban was lifted. At the same time, 3.7% of pre-ban smokers continued smoking during the ban, but quit after the ban was lifted.

Table 2 provides descriptive statistics of smoking prevalence, for males and females, directly before, during and after the sales ban. As in many countries, smoking prevalence in South Africa is substantially higher among males than among females. The decrease in smoking prevalence among males (9.2%, from 28.0% to 25.6%) during the ban is slightly, but insignificantly, less than the decrease in smoking prevalence among females (11.6%, from 6.9% to 6.1%). After the ban was lifted, smoking prevalence increased marginally for both sexes.

### Analysis of the two surveys, applying a longitudinal approach

In this part of the study, we exploit the longitudinal aspects of the data by focusing on the 480 respondents who indicated in the two separate surveys that they smoked in both 2017 and in the week before the sales ban was implemented (Table 3). We track individuals over time through their personal identifier, provided by the NIDS data.

The smokers captured in Table 3 are likely to be more ‘established’ smokers (i.e. people who have been smoking for at least three years). Comparing Tables 1 and 3 thus allows us to assess how smoking behavior might differ between ‘established’ smokers and all smokers (i.e. the ‘established’ smokers, plus those who either started smoking between 2017 and 2020, or who gave false information in either of the two surveys). Of the established smokers, 7% quit during the sales ban (Table 3), compared to a 7.8% quitting rate amongst all smokers (Table 1).

More than 90% of established smokers continued smoking cigarettes during the tobacco sales ban. After the sales ban, nearly 95% of pre-ban established smokers indicated that they were still smoking cigarettes; 71% (5.0%/7.0%) of established smokers who quit during the sales ban indicated that they had relapsed after the ban was lifted. Only 2% of pre-ban established smokers indicated that they smoked neither during the sales ban nor after the lifting of the ban. After the sales ban was lifted, 2.4% of established smokers quit smoking.

## DISCUSSION

South Africa was one of only three countries (alongside India and Botswana) that implemented a sales ban as part of its response to the COVID-19 pandemic. It was one of the most controversial aspects of the country’s COVID-19 lockdown. Several studies^10-14^ were conducted during the sales ban to determine its impact on smoking behavior. The two most comprehensive studies were not based on nationally representative data^12,13^, and the other two did not provide sufficient information to support their claim that their surveys were, in fact, nationally representative^10,11^.

We used two approaches to investigate smokers’ quitting behavior. The first approach compared the descriptive statistics of the smoking population before, during and after the sales ban. The second approach used the longitudinal aspects of a subset of NIDS wave 5, and NIDS-CRAM data. Both approaches indicated that the sales ban has had a limited impact on smokers’ quitting behavior. Between 7% and 8% of pre-lockdown smokers indicated that they had quit during the sales ban. This decreases South Africa’s smoking prevalence by 1.4 percentage points. To put this into context, between 1993 and 2000 smoking prevalence in South Africa decreased by an average of 0.8 percentage points per year^22^. These decreases were driven by sharp increases in the excise tax (more than 10% above the inflation rate). The reduction in smoking prevalence achieved during the sales ban is thus equivalent to less than two years’ worth of sharp excise tax increases.

We found two studies that assessed the impact of the tobacco sales ban in India^23,24^ but none in Botswana. Based on a sample of 801 respondents in two cities (Delhi and Chennai), one of the Indian studies found that 11.3% of tobacco users stopped using tobacco during India’s lockdown^23^. At first sight, this is a somewhat higher quitting percentage than in South Africa, but the study does not indicate whether the quitters used smoked and smokeless tobacco, so it is not directly comparable to the current study. Unsurprisingly, however, the study finds that tobacco users that reported that they had access to tobacco products were less likely to quit than those who reported that they had no access.

The second Indian study, based on a sample of 650 participants enrolled in a tobacco cessation program before the lockdown, found that 38% of tobacco users abstained from using tobacco after the start of the lockdown^24^. While the proportion of quitters is substantially higher than that found in the other India study^23^ and the present study, the results are not comparable because it only included participants who were already motivated to quit because they were enrolled in a cessation program. However, as is the case in South Africa, the analysis indicates that tobacco products were mostly available, despite the sales ban^24^.

Studies conducted in South Africa gave substantially different estimates of the percentage of smokers that quit during the sales ban period. At one extreme, a survey of just more than 2000 smokers by a technology company, M4JAM, indicates that 49% of respondents had quit smoking during the sales ban^11^. At the other extreme, two surveys (n=12204 and n=23631, respectively) by REEP found that 7.4% of smokers in the sample had quit smoking by May 2020, and that 9.0% of smokers in the sample had quit smoking by June 2020^13,14^. A survey by a market research company, ‘Ask Afrika’ (n=1412), found that 18% of respondents indicated that they smoked before the sales ban and only 10% reported that they smoked during the sales ban, which means that 44% of smokers quit during the ban^10^.

M4JAM and ‘Ask Afrika’ claim that the samples are representative of the South African population. The REEP studies explicitly indicate that they are not nationally representative. Despite the drawbacks of the REEP studies, their estimates of quitting during the sales ban period correspond very closely to the estimates in this broadly nationally representative survey.

Before the sales ban was implemented in South Africa, the country already struggled with high levels of illicit trade^6^. Illicit market channels were well-established in the informal market^7^. The sales ban was very effective in shutting down formal retailers as a channel through which smokers could purchase cigarettes^18^. However, the ban proved a boon to informal tobacco outlets, which were able to sell increased quantities of cigarettes at highly inflated prices to desperate smokers^18^.

To implement a tobacco sales ban in a situation where the illicit market was already well-entrenched was problematic. Legal sales volumes in the 2020–2021 financial year were 48% lower than in the previous year (1 March 2019 to 29 February 2020). In the 2021–2022 financial year, i.e. when the sales ban was no longer in place, legal sales volumes recovered somewhat, but were still 42% below the legal sales volumes of the 2019–2020 financial year. The decrease in smoking prevalence between 2019 and 2021 explains only a modest proportion of the decrease in legal sales. The numbers clearly imply that the illicit market increased substantially between 2019 and 2021. A recent independent study has shown that the illicit market comprised more than 50% in 2020, and remained at that level in 2021, despite the fact that the cigarette market has been normalized after the lifting of the sales ban^25^. This has negative implications for government revenue and public health.

With the benefit of hindsight, one can argue that the sales ban was misguided. Yet in the early days of the pandemic there were huge uncertainties. The South African government’s precautionary approach thus makes sense. However, the extension of the sales ban beyond the first five weeks of the lockdown seems unjustified. Within weeks of the start of the sales ban, the media reported on the wide availability of illicit cigarettes. A research report^13^, based on an online survey of more than 12000 respondents, published seven weeks after the start of the sales ban, indicated clearly that most smokers did not quit and were able to purchase cigarettes on the illicit market. This report received a lot of media coverage and was shared with members of the National Coronavirus Command Council (NCCC). The NCCC had been set up by the President to manage the country’s response to the pandemic.

A subsequent research report^12^, published 16 weeks after the start of the sales ban, indicated that most smokers who quit during the sales ban did so in the first four or five weeks. After the first five weeks, the number of quitters dropped to a trickle. Despite all the evidence that the tobacco sales ban was being bypassed on a large scale, the government kept it in place for another four weeks.

In the current analysis, we made the unexpected finding that 3.7% of pre-ban smokers quit smoking after the sales ban was lifted. This may have been because the tobacco sales ban was implemented in a very stressful period, which would have made it more difficult for the average smoker to quit. As time progressed and people became more accustomed to living in the pandemic, the need for stress releasers like cigarettes may have dwindled, leading some people to quit, even though cigarettes, at that stage, were legally available. It might well be that the coercion of the sales ban was counterproductive; most smokers did not respond in the way that the government expected them to do.

That the sales ban may have been counterproductive is supported by evidence from the US^26^ and the UK^27^. Neither of these countries imposed bans on the sale of tobacco, yet research has documented declines in cigarette smoking during the early stages of the pandemic in both of these countries. For example, a longitudinal analysis of a sample of young smokers in the US found that between 2019 and 2020, participants had 40% lower odds of reporting past 30-day cigarette use^26^. Reasons offered for this include the fact that lockdowns reduced opportunities for smoking in social settings, decreased people’s access to retailers, and caused changes to people’s habits of purchasing tobacco such as stopping to purchase cigarettes on the way to work.

Overall, the outcome of the sales ban in South Africa, in terms of the proportion of people who quit cigarette smoking, is disappointing. In fact, fewer than 4% of pre-ban smokers maintained an extended period (of at least 7–8 months) of abstinence after having quit during the sales ban period. For cigarette smokers who had been smoking both in 2017 and just before the start of the sales ban, this figure is only 2%.

The process of quitting smoking is complex and there is no one-size-fits-all quitting mechanism^28^. While a sales ban conceptually may create the right conditions to encourage people to quit, the South African experience shows that if there is a demand for cigarettes, the supply will be forthcoming. This is especially the case when the sales ban is implemented in the context of high levels of illicit trade. A large body of evidence also provides insight into the reason why the demand for cigarettes did not simply fall following the introduction of the tobacco sales ban; it is very difficult to quit smoking^28-31^.

Best-practice policy for the development of a national-level smoking cessation program is enshrined in the World Health Organization Framework Convention on Tobacco Control (WHO FCTC)^32^, which South Africa ratified in 2005. According to the treaty and its corresponding implementation guidelines^33^, a comprehensive smoking cessation program should include behavioral therapy and pharmacological support for smokers. It should also include a national toll-free cessation hotline, subsidized support in health facilities and subsidized nicotine replacement therapy^33^.

While smoking cessation counselling is available in South Africa through various quit line services provided by private organizations, there is no toll-free national ‘quitline’ in the country^34^. Pharmacological support, in the form of nicotine replacement therapy, is not subsidized and smoking cessation medications are more expensive than tobacco products^34^. In addition to the fact that cessation support to smokers is limited in South Africa, the government did not launch any information campaigns to notify smokers of the existing cessation support services during the tobacco sales ban. The disappointing cessation figures reported in this study are therefore not entirely unexpected.

### Limitations

This study has limitations. Firstly, because we were allocated only 60 s to ask all tobacco-related questions in the survey, we could ask only a few questions. For example, we were unable to ask about respondents’ smoking behavior (e.g. smoking intensity) prior to the lockdown. We did not ask any questions about non-cigarette tobacco products. Also, the questionnaire did not explicitly ask whether smokers quit during the sales ban; this was derived from the daily number of cigarettes smoked during that time. It is possible that smokers who quit at some point during the sales ban may report average cigarette consumption (which would be a non-zero number). This would understate the number of quitters during the sales ban. However, the evidence from another survey^12^ indicates that most quitting happened in the first few weeks of the sales ban, suggesting that this should not bias our results significantly. Secondly, the NIDS-CRAM sample was not as large as one would have hoped for in an ideal circumstance, which results in less precise prevalence estimates. Females were substantially oversampled. The weighting mostly corrected this, but even in the weighted sample, females are slightly over-represented.

## CONCLUSIONS

This study provided the first broadly nationally representative view of the impact of South Africa’s temporary tobacco sales ban in 2020 on smoking prevalence. Results showed that, despite the unprecedented nature of the sales ban, and its disruptiveness, less than 8% of smokers quit in response to the ban, and most smokers continued smoking. More than half of smokers who quit during the ban relapsed after the ban was lifted. The fact that cessation support in South Africa is limited and that the government did not provide smokers with any information on how to quit smoking during the ban are policy failures that may explain the low quitting rates of South African smokers in response to the sales ban.

The sales ban was also implemented in a period when the illicit cigarette market was already well entrenched. The market in illicit cigarettes boomed during the sales ban. Even after the sales ban was lifted, the legal market remains substantially smaller than it was in early 2020. The increase in the illicit market is an unfortunate, but predictable, consequence of South Africa’s tobacco sales ban.

## Data Availability

The data supporting this research are available from the following link: https://www.datafirst.uct.ac.za/dataportal/index.php/catalog/851

## Footnotes

a The interested reader is referred to the NIDS-CRAM technical reports available on https://cramsurvey.org/reports/#wave-1 for further information.

## References

[1] Government of South Africa (2020). Disaster Management Act, 2002: Amendment of Regulations Issued in terms of Section 27(2).

[2] Southern African Legal Information Institute (SAFLII) (2020). Fair-Trade Independent Tobacco Association v President of the Republic of South Africa and Another (21688/2020) [2020] ZAGPPHC 246.

[3] Nicolson G (2020). Tobacco ban: Unpacking Dlamini Zuma’s defence.

[4] Bonevski B, Bialek C, Skelton E (2020). Smoking increases your coronavirus risk. There’s never been a better time to quit.

[5] British Heart Foundation Smoking. HeartMatters.

[6] Vellios N, van Walbeek C, Ross H (2020). Illicit cigarette trade in South Africa: 2002-2017. Tob Control.

[7] van der Zee K, Vellios N, van Walbeek C, Ross H (2020). The illicit cigarette market in six South African townships. Tob Control.

[8] van Walbeek CP, Filby S (2021). Close, but no cigar: Excise tax on cigarettes should have been increased by 50%.

[9] Financial Standing Committee of South Africa (2019). Illicit Tobacco Trade: SARS briefing.

[10] Ask Afrika (2020). The ask Afrika COVID-19Tracker: week 12 results, level 3 advanced.

[11] Suid-Kaap Forum (2020). Lockdown is a new dawn for nearly half of South African smokers, 2020.

[12] van Walbeek C, Filby S, van der Zee K (2020). Smoking and Quitting Behaviour in Lockdown South Africa: Results from a second survey.

[13] van Walbeek CP, Filby S, van der Zee K (2020). Lighting Up The Illicit Market: Smoker’s Responses to the Cigarette Sales Ban in South Africa.

[14] van Walbeek CP, van der Zee K, Filby S (2020). Smoking and Quitting Behaviour in South Africa After the Tobacco Sales Ban: Results From A Third Survey.

[15] Human Sciences Research Council (2020). Majority of South Africans adhere to lock down regulations affecting the sale of tobacco products.

[16] Saloojee Y, Mathee A (2022). COVID-19and a temporary ban on tobacco sales in South Africa: impact on smoking cessation. Tob Control.

[17] Egbe CO, Ngobese SP (2020). COVID-19lockdown and the tobacco product ban in South Africa. Tob Induc Dis.

[18] Filby S, van der Zee K, van Walbeek C (2022). The temporary ban on tobacco sales in South Africa: lessons for endgame strategies. Tob Control.

[19] Ingle K, Brophy T, Daniels R (2020). National Income Dynamics Study – Coronavirus Rapid Mobile Survey (NIDS-CRAM) panel user manual. Version 2.

[20] Ardington C (2020). NIDS-CRAM Wave 1 Data Quality. Version 2.

[21] Ramaphosa C (2021). President Cyril Ramaphosa: Response to debate on State of the Nation Address 2021.

[22] van Walbeek C (2002). Recent trends in smoking prevalence in South Africa--some evidence from AMPS data. S Afr Med J.

[23] Arora M, Nazar GP, Sharma N (2022). COVID-19 and tobacco cessation: lessons from India. Public Health.

[24] Gupte HA, Mandal G, Jagiasi D (2020). How has the COVID-19pandemic affected tobacco users in India: lessons from an ongoing tobacco cessation program. Tob Prev Cessat.

[25] Vellios N (2022). How big is the illicit cigarette market in South Africa?.

[26] Denlinger-Apte R, Suerken CK, Ross JC (2022). Decreases in smoking and vaping during COVID-19stay-at-home orders among a cohort of young adults in the United States. Prev Med.

[27] Kale D, Perski O, Herbec A, Beard E, Shahab L (2022). Changes in cigarette smoking and vaping in response to the COVID-19pandemic in the UK: findings from baseline and 12-month follow up of HEBECO study. Int J Environ Res Public Health.

[28] Borland R, Hyland A, Cummings KM, Fong GT (2010). One size does not fit all when it comes to smoking cessation: observations from the International Tobacco Control Policy Evaluation Project. Nicotine Tob Res.

[29] Marlatt GA, Curry S, Gordon JR (1988). A longitudinal analysis of unaided smoking cessation. J Consult Clin Psychol.

[30] Piasecki TM, Fiore MC, McCarthy DE, Baker TB (2002). Have we lost our way? The need for dynamic formulations of smoking relapse proneness. Addiction.

[31] Herd N, Borland R, Hyland A (2009). Predictors of smoking relapse by duration of abstinence: findings from the International Tobacco Control (ITC) Four Country Survey. Addiction.

[32] World Health Organization Framework Convention on Tobacco Control (2003). WHO Framework Convention on Tobacco Control.

[33] World Health Organization Framework Convention on Tobacco Control (2013). Guidelines for implementation: article 14.

[34] World Health Organization (2021). WHO report on the global tobacco epidemic 2021: addressing new and emerging products.

